# Supplementary material for: Oligodendroglia-to-pericyte conversion after lipopolysaccharide exposure is gender-dependent
Source: PLoS One. 2024 Aug 6;19(8):e0308132. doi: 10.1371/journal.pone.0308132 (PMC11302866; doi:10.1371/journal.pone.0308132)
Supplement: Attachment — Submitted filename: Response to Reviewers.docx [file pone.0308132.s002.docx]

**Editor Comments:**

1. Please note that funding information should not appear in any section or other areas of your manuscript. We will only publish funding information present in the Funding Statement section of the online submission form. Please remove any funding-related text from the manuscript.

*Ans：We have removed funding-related text from the manuscript.*

2. Thank you for uploading your study's underlying data set. Unfortunately, the repository you have noted in your Data Availability statement does not qualify as an acceptable data repository according to PLOS's standards.

*Ans：We have re-uploaded the data to the BioStudies. The link is https://www.ebi.ac.uk/biostudies/studies/S-BSST1487*

**Editor Comments:**

1. Please ensure that your manuscript meets PLOS ONE's style requirements, including those for file naming.

*Ans: We have adjusted the formatting according to the guidelines.*

2. Please state what role the funders took in the study.  If the funders had no role, please state: ""The funders had no role in study design, data collection and analysis, decision to publish, or preparation of the manuscript."" If this statement is not correct you must amend it as needed. Please include this amended Role of Funder statement in your cover letter; we will change the online submission form on your behalf.

*Ans: We have added the role of funder statement in the cover letter.*

3. Please upload a copy of Supporting Information Figure/Table/etc. "S1 Data. Raw data of this article" which you refer to in your text on page 23 in PDF submission.

*Ans: We have uploaded the “S1_Data sheet (XLSX)” of this article” and the raw data is stored in the BioStudies.*

*The link is https://www.ebi.ac.uk/biostudies/studies/S-BSST1487*

4. Please review your reference list to ensure that it is complete and correct. If you have cited papers that have been retracted, please include the rationale for doing so in the manuscript text, or remove these references and replace them with relevant current references. Any changes to the reference list should be mentioned in the rebuttal letter that accompanies your revised manuscript. If you need to cite a retracted article, indicate the article’s retracted status in the References list and also include a citation and full reference for the retraction notice.

*Ans: We have removed the reference:* *Montagne A, Nikolakopoulou AM, Zhao Z, Sagare AP, Si G, Lazic D, et al. Pericyte degeneration causes white matter dysfunction in the mouse central nervous system. Nat Med. 2018;24(3):326-37. doi: 10.1038/nm.4482.*

**Additional Editor Comments:**
Reviewer #1: The research article entitled “Gender-specific oligodendroglia-to-pericyte conversion following lipopolysaccharide administration” is interesting in terms of its novelty in exploring the differentiation of oligodendroglia in different sexes and pathological conditions.

1. However, the article has major shortcomings in the presentation. It lacks the proper flow and clear explanations. The introduction is poorly written. The authors have mentioned many sentences that are not necessary and, on the contrary, didn’t include the information that is required in the manuscript.

*Ans：Thank you very much for the suggestion. We have revised and re-written the manuscript to make it clear and straightforward.*

2. In the abstract section authors have mentioned that conversion of female Sox10 cells to pericytes was significantly higher than that of males when exposed to LPS. In the following sentence, they mention the conversion rate of RFP cells is higher in the untreated female and the LPS-treated male group, with the latter not significantly different from the untreated male group. The authors have not mentioned the difference between the Sox10 cells and RFP cells and have used them interchangeably. If they meant it to be the same, why are the authors mentioning the conversion rate of RFP cells is higher in the LPS-treated male group? It doesn't need to be mentioned when it's significantly not different from the untreated male group. Also, is it so important to mention it in the abstract, and where in the main data they have shown it?

*Ans：Thank you for the helpful suggestion. We have deleted the incorrect sentences and revised the manuscript. Please see the revision.*

3. In the results section 3.1, what does low-dose condition mean? It’s not clearly mentioned. The heading says females have more Sox10-A cells while it should be “females have more conversion of Sox10-A to pericytes”.

*Ans：We have corrected this in the manuscript.*

4. Support lines 252-255 with references.

*Ans: We have removed the paragraphs to enhance the clarity of the manuscript and discussed it in the revision.*

5. Authors have shown the conversion of Sox10 cells to pericyte on exposure to LPS which is significantly higher in females compared to males. In the discussion section, the authors mention the loss of pericyte on exposure to LPS. Authors should clearly explain this disparity/confusion in the discussion section.

*Ans: We have added new data on propidium Iodide labeling of necrotic cells to the article as Fig.5.*

6. Overall, the manuscript needs rewriting to present the data explaining every relevant thing clearly and explicitly in detail before further consideration.

*Ans: We have re-written the manuscript according to the reviewer’s suggestion.*

Reviewer #2: The study explores sex-specific differences in the conversion of Sox10 cells into pericytes and its implications for blood-brain barrier integrity. Female mice show enhanced differentiation compared to males. The findings highlight the importance of considering sex-specific responses in addressing blood-brain barrier disruptions in neurodegenerative conditions.

1. Could the authors provide more insight into the mechanisms underlying the observed sex-specific differences in the conversion of Sox10 cells into pericytes, particularly regarding the role of Erα and potential interactions with other signaling pathways?

*Ans: We have discussed the potential interactions with cholesterol metabolism in the article. Please see the discussion.*

2. In light of the contrasting findings with previous studies on Sox10 cell differentiation into neurons and astrocytes, what factors do the authors believe might account for these discrepancies, and how could future research reconcile these differences?

*Ans: We think high doses of tamoxifen may cause neuronal injury and further induce the conversion of Sox10 cells to neurons. We have discussed this in the revision. Regarding previous studies on the conversion of OPCs to astrocytes, we believe more biomarkers like ALDH1L1, Sox9, and AQP4 should be used to confirm astrocytes instead of relying solely on GFAP.*

3. Can the authors elaborate on the potential clinical implications of their findings regarding the influence of low-dose tamoxifen treatment on Sox10 cell differentiation pathways, particularly in the context of enhancing blood-brain barrier repair and slowing the progression of neurodegenerative diseases?

*Ans: We have discussed this in the revision.*

4. Considering the limitations mentioned in the article, such as potential confounding factors, could the authors discuss how these limitations might have affected the interpretation of the results and suggest strategies for addressing them in future studies?

*Ans: We suggest using tamoxifen-injected Cre mice of the same gender as controls, rather than just wild-type controls. We have discussed it in the manuscript.*

5. How do the findings of this study contribute to our understanding of pericyte loss in neurodegenerative diseases like Alzheimer's disease and stroke, and what avenues for further research do the authors believe should be explored to build upon these findings?

*Ans: Pericyte loss may be related to the formation of amyloid β in Alzheimer's disease and disruption of interpericyte tunnelling nanotubes (IP-TNTs) in stroke. We have discussed it in the revision.*

6. Are there any specific experimental approaches or analyses that the authors believe would strengthen the conclusions of the study or provide additional insights into the mechanisms underlying sex-specific responses in Sox10 cell differentiation?

*Ans: Thank you for your suggestion. We have added new data on propidium Iodide labeling of necrotic cells after LPS injection to the article, presented as Fig.5.*
